# Endoplasmic Reticulum Stress in Macrophages: The Vicious Circle of Lipid Accumulation and Pro-Inflammatory Response

**DOI:** 10.3390/biomedicines8070210

**Published:** 2020-07-13

**Authors:** Vasily N. Sukhorukov, Victoria A. Khotina, Mariam Bagheri Ekta, Ekaterina A. Ivanova, Igor A. Sobenin, Alexander N. Orekhov

**Affiliations:** 1 Research Institute of Human Morphology, Laboratory of Cellular and Molecular Pathology of Cardiovascular System, 3 Tsyurupy Str., 117418 Moscow, Russia; nafany905@gmail.com (V.A.K.); ms.bvgheri@gmail.com (M.B.E.); igor.sobenin@gmail.com (I.A.S.); a.h.opexob@gmail.com (A.N.O.); 2 Laboratory of Angiopathology, Institute of General Pathology and Pathophysiology, 8 Baltiyskaya Str., 125315 Moscow, Russia; 3 Institute for Atherosclerosis Research, Skolkovo Innovative Center, 121609 Moscow, Russia; 4 Laboratory of Medical Genetics, National Medical Research Center of Cardiology, Institute of Experimental Cardiology, 15-a 3-rd Cherepkovskaya Str., 121552 Moscow, Russia

**Keywords:** atherosclerosis, endoplasmic reticulum stress, macrophages, pro-inflammatory response, foam cells

## Abstract

The endoplasmic reticulum (ER) stress is an important event in the pathogenesis of different human disorders, including atherosclerosis. ER stress leads to disturbance of cellular homeostasis, apoptosis, and in the case of macrophages, to foam cell formation and pro-inflammatory cytokines production. In atherosclerosis, several cell types can be affected by ER stress, including endothelial cells, vascular smooth muscular cells, and macrophages. Modified low-density lipoproteins (LDL) and cytokines, in turn, can provoke ER stress through different processes. The signaling cascades involved in ER stress initiation are complex and linked to other cellular processes, such as lysosomal biogenesis and functioning, autophagy, mitochondrial homeostasis, and energy production. In this review, we discuss the underlying mechanisms of ER stress formation and the interplay of lipid accumulation and pro-inflammatory response. We will specifically focus on macrophages, which are the key players in maintaining chronic inflammatory milieu in atherosclerotic lesions, and also a major source of lipid-accumulating foam cells.

## 1. Introduction

Atherosclerosis is a chronic disease, which is associated with lipid accumulation and inflammation in large and medium-sized arteries [1]. Macrophages are regarded as key players in atherosclerosis development providing for a large part of intracellular lipid accumulation in the plaque and for propagation of the pro-inflammatory response. Modified particles of low-density lipoprotein (LDL) are taken up by macrophages through unspecific phagocytosis, a process associated with increase of pro-inflammatory cytokines release [2]. Macrophages, as well as other cell types of the arterial wall, change their appearance upon lipid accumulation, becoming so-called foam cells. Although these processes are currently well documented, the exact mechanisms that mediate foam cell formation and pro-inflammatory cytokines release, remain to be fully understood. One of the possible mechanisms, which underlies macrophage lipid accumulation and inflammatory response, is endoplasmic reticulum (ER) stress. ER stress has been reported in numerous human pathologies [3]. It is characterized by profound disturbance of the ER functioning, calcium (Ca^2+^) signaling, and protein synthesis and is associated with such atherosclerosis-driven factors as apoptosis [4], efferocytosis [5], foam cell formation [6], and inflammation [7]. However, the relationships of these factors still need to be clarified. ER stress can affect virtually any cell with active metabolism and protein synthesis. In atherosclerosis, the major cell types involved in the disease development are macrophages, endothelial cells, smooth muscle cells, and pericytes. All these cell types can present with signs of ER stress in atherosclerotic lesions at different stages of development. In this review, we will specifically focus on macrophages, since these cells play a key role in maintaining pro-inflammatory conditions in atherosclerotic lesions, and are also the major source of foam cells that are responsible for lipid accumulation in the plaque.

The endoplasmic reticulum is a complex cytoplasmic membrane structure, which is present in all eukaryotic cells [8]. The ER is involved in protein synthesis, folding, and modification, lipid synthesis, sensing and trafficking, and also, intracellular Ca^2+^ balance regulation. ER stress associated with unfolded protein response (UPR) may lead to the induction of inflammatory response, lipid accumulation, and/or apoptosis [9]. The classical mechanism, which leads to activation of ER stress-related UPR pathways is accumulation of unfolded and misfolded proteins in the ER lumen [10]. Normally, UPR plays a compensatory role in reducing the ER stress through blocking protein synthesis, which prevents further accumulation of misfolded proteins in the ER, up-regulation of chaperone translation, and degradation of misfolded proteins. However, upon prolonged ER stress, when the adaptive UPR cannot restore homeostasis, it can trigger apoptosis. The main mechanism of apoptosis induction in UPR is expression of the transcription factor CCAAT/enhancer-binding protein-homologous protein (CHOP) and Jun amino-terminal kinase (JNK) kinase [11,12,13]. The UPR includes three signaling pathways that are activated by three ER- resident proteins, namely protein kinase RNA-like ER kinase (PERK), inositol requiring protein 1α (IRE1α), and activating transcription factor 6 (ATF6). All three proteins have ER lumen domains inactivated by association with the GRP78/Bip chaperone. Under ER stress, GRP78/BiP dissociates from these proteins that triggers activation of three different UPR signaling pathways [14].

The activated PERK serine-threonine kinase launches the first UPR pathway by phosphorylation of the α-subunit of eukaryotic initiation factor 2 (eIF2α) that leads to the arrest of protein translation and induction of selective mRNAs translation, including activating transcription factor 4 (ATF4). Generally, ATF4 plays an important role in regulating a wide range of genes involved in cellular adaptation to stress. However, under prolonged ER stress, ATF4 can induce the expression of CHOP, which is the main inducer of apoptosis [15]. As a transcription factor, CHOP interacts downstream with oxidase ER oxidoreduclin 1α (ERO1α), which induces overexpression of proapoptotic proteins PUMA, BAX, and Caspase-3 and decreases expression of BCL-2, thereby promoting caspase-dependent macrophage apoptosis [16,17]. In addition, CHOP plays an important role in switching between autophagy and apoptosis, which is described in detail in the recent review [18].

In the IRE1α-mediated UPR pathway, IRE1α specifically splices mRNA encoding X-box binding protein 1 (XBP1), which promotes chaperons expression [19]. IRE1α may also contribute to the cleavage of other ER-localized mRNAs and miRNAs, leading to their degradation through regulated Ire1-dependent decay (RIDD). RIDD may participate in the regulation of homeostasis, cell survival adaptation, and apoptosis [20]. Similar to PERK, IRE1α can trigger apoptosis under prolonged ER stress. IRE1 associated with the adaptor protein tumor necrosis factor (TNF) receptor-associated factor (TRAF)2. IRE1α/TRAF2 pathway induces a pro-apoptotic signaling pathway through caspase-12 activation [21]. Another pro-apoptotic signaling pathway of IRE1/apoptosis-signaling-kinase 1 (ASK1) is mediated by the induction of JNK and p38 mitogen-activated protein kinase (MAPK), which activate CHOP [22,23].

Finally, ATF6 cleavage in the Golgi complex by specific site-1 and site-2 proteases activates another distinct UPR pathway [24]. Activated ATF6 translocates to the nucleus and mediates the expression of XBP1 genes, as well as nuclear factor kappa-light-chain-enhancer of activated B-cell (NF-κB) transcription factor, and CHOP. NF-κB induces the expression of various pro-inflammatory cytokines, such as TNF-α and interleukin (IL)-6, leading to pro-inflammatory response [22].

## 2. The Role of ER Stress in the Formation of Foam Cells

One of the causes of the ER stress development in macrophages is the excessive free cholesterol accumulation in the ER. In atherosclerosis, macrophages actively uptake oxidized and other modified atherogenic low-density lipoprotein (LDL) particles. Internalized LDL is processed in lysosomes, releasing cholesterol esters and triglycerides, which are converted to fatty acids and free cholesterol by lysosomal acid lipase (LAL) and then transported to the ER [25,26]. Direct relationship between intracellular lipid metabolism and ER stress may be mediated by cholesterol sensor proteins, which are localized in the ER membrane [27]. Moreover, ER stress may be triggered by an excessive amount of lipids because of their low level in the ER membrane under normal conditions. It has been shown that transmembrane domains of IRE1 and PERK may be sensitive to the lipid composition of the ER membrane bilayer. Thus, high amounts of exogenous lipids can change the biophysical properties of the ER membrane and launch the UPR by activation of PERK and IRE1 in the lipid bilayer [28]. Furthermore, various stress factors, including oxidized LDL (oxLDL) and oxysterols, can negatively affect the process of protein folding in the ER by perturbations of the ER lipid bilayer composition, which leads to UPR activation [29,30]. It has been also shown that modified atherogenic LDL, such as oxLDL, can cause up-regulation of scavenger receptors such as SR-A, CD36, and lectin-like oxidized LDL receptor-1 (LOX-1) via activation of ER stress response [31,32,33]. CD36 triggers signaling pathways that activate MAP kinases, Src family kinases, and Vav family guanine nucleotide exchange factors, leading to ligand internalization and foam cell formation [33,34]. CD36 is currently regarded as a possible key receptor in foam cells formation, since CD36^-/-^ macrophages did not accumulate cholesterol esters upon incubation with oxLDL [35]. Toll-like receptor 4 (TLR4) can also mediate cholesterol accumulation and subsequent activation of ER stress in macrophages, which were challenged by modified LDL. In this process, TLR4 may mediate ER stress through IRE1 and ATF6 pathways [36]. Thus, influx of cholesterol is increased in macrophages during ER stress.

Another important feature of foam cell formation is reduced expression of ATP-binding cassette transporter A1 (ABCA1) and G1 (ABCG1) genes, and scavenger receptor class B type I (SR-BI) under activation of UPR pathways, which interferes with cholesterol efflux [37]. Cholesterol transfer from macrophages to high density lipoprotein-cholesterol (HDL) through the ABC transmembrane transporters can decrease cellular cholesterol concentration [38,39]. ABC transporters’ expression is tightly regulated by genetic (transcription factors) and epigenetic factors (miRNAs and RNA-binding proteins) in macrophages under physiology conditions [40,41]. Downregulation of miR-21 expression and subsequent activation of p38MAPK/CHOP and JNK signaling pathways mediated by UPR can contribute to efflux. CHOP can inhibit expression of ABCG1 and SR-BI. Increased regulatory miRNAs expression, in particular miR-33 also affect lipid metabolism [42]. It was suggested that ATF6 could participate in lipoprotein metabolism by activating the PI3K/Akt signaling pathway, which inhibits ABCA1 and ABCG1 genes expression in macrophages, and a subsequent decrease in cholesterol efflux [43]. Moreover, oxLDL in macrophages was shown to activate two other UPR signaling pathways—PERK/eIF2α/ATF4 and IRE1α/XBP1 [37]. Activation of the transcription factor ATF4 can suppress the ABCA1 expression and decrease cholesterol efflux [44]. MiR-33 is involved in the regulation of HDL metabolism, cholesterol efflux, and fatty acid oxidation by downregulating ABC transporter expression [45]. During ER stress, mirR-33 overexpression can lead to a decrease of ABCA1 levels in macrophages and further lipid accumulation [42]. Thus, imbalance of ABCA1 and ABCG1 genes expression as a result of intracellular events caused by ER stress, may promote foam cell formation.

ER stress may also contribute to lipid accumulation in macrophages by controlling the activity of sterol regulatory element binding proteins 1 and 2 (SREBP1 and 2). SREBPs are ER-resident transcription factors that regulate the expression of genes encoding LDLR and proteins involved in cholesterol and fatty acid biosynthesis [22]. Inhibition of GSK3α/β kinases reduces cholesterol uptake caused by ER stress and the expression of genes involved in the regulation of lipid and cholesterol metabolism, such as fatty acid synthase (FAS), SREBP1c, SREBP2, 3-hydroxy-3-methylglutaryl-coenzyme A (HMG -CoA) reductase, and LDLR [46]. Interaction of ATF6 and SREBP2 can lead to increased expression of cholesterol biosynthesis genes, including HMG-CoA reductase and HMG-CoA synthase [47]. It has long been known that PERK regulates the expression of key enzymes of intracellular lipid metabolism, including FAS. Normally, PERK can mediate lipid synthesis by activation of SREBP1 through the eIF2-dependent decrease the level of the regulatory protein Insig1 [48].

Thus, ER stress regulates the lipid metabolism in macrophages by stimulating cholesterol uptake, inhibiting cholesterol efflux, and regulating the expression of cholesterol membrane transporters, which can lead to intracellular lipid accumulation in macrophages and their subsequent differentiation into foam cells (Table 1).

## 3. The Role of ER Stress in Apoptosis

Under prolonged ER stress, UPR may launch apoptosis which might be provoked by excessive lipid accumulation or pro-inflammatory stimulation of cells. High concentrations of free cholesterol, oxysterols, and oxLDL cause activation of the ER stress-mediated pathway, which promotes macrophage apoptosis via CHOP activation [49]. A significant increase in CHOP expression was found in atherosclerotic plaques macrophages, indicating of ER stress [10]. Excess saturated fatty acids and free cholesterol in the ER contribute to the ER stress and activation of all three UPR pathways, which can subsequently lead to apoptosis via activation of CHOP through IRE1α/JNK/MAPK, IRE1α/XBP1, ATF6/XBP1, and PERK/eIF2α/ATF4 pathways [18,50].

In addition, it has been reported that exposure to oxLDL resulted in a slight increase of cytosolic Ca^2+^ level through a mechanism, which remains to be elucidated, but is likely to involve ER stress. Such increase of cytosolic Ca^2+^ may act as a ER stress-associated macrophages apoptosis inducer [51]. The main pool of cellular Ca^2+^ is localized in the ER cisterns, and increased calcium level in cisterns and cytoplasm can lead to absorption of Ca^2+^ by mitochondria, which subsequently leads to apoptosis [52]. However, a decreased calcium release and, accordingly, the influx of Ca^2 +^ in macrophages also causes ER stress, increased proteins expression and, as a result, apoptosis [53].

Accumulating evidence demonstrates the important role of HDL in the macrophage apoptosis in atherosclerotic lesions [54]. The increased macrophage apoptosis in atherosclerotic lesions was detected in mice with LDLR^-/-^ and post-synaptic density protein / Drosophila disc-large protein / Zonula occludens protein containing 1 (PDZK1) deficiency in leukocytes. PDZK1 is an adapter protein that binds to SR-BI (HDL receptor). Under physiological conditions, HDL was shown to reduce macrophage apoptosis caused by ER stress. However, the opposite effect was observed in PDZK1-deficient macrophages. This may explain the role of this adapter protein in atherogenesis and apoptosis of macrophages [54]. For the first time, it was also shown that oxidized HDL (oxHDL), unlike native HDL, can activate macrophage apoptosis by inducing the ER stress/CHOP pathway through enhanced oxidative stress, which can be mediated by interaction of oxHDL with TLR4 [55]. In addition, suppression of the ER stress/CHOP pathway in macrophages caused by oxLDL could be achieved by stimulating the expression of apolipoprotein A-I [29]. Sphingosine-1-phosphate (S1P) is one of the components of HDL, which has an anti-apoptotic effect and promotes alternative (anti-inflammatory) polarization of macrophages, and an important player in various processes in macrophages [56]. S1P also has an atheroprotective effect during the progression of atherosclerosis. The antiatherogenic effect of S1P may partially depend on specific action of sphingosine 1 phosphate receptor 1-4 (S1PR1-4) in various types of cells [57]. It was shown that activation of S1PR1, which is a G-protein receptor, and presence of HDL, which is the main carrier of S1P, can protect macrophages from apoptosis. The signaling pathways PI3K/Akt and JAK2/STAT3 appear to play an important role in this process [58].

Inflammatory response can enhance apoptosis in atherosclerotic lesion macrophages. It was shown that IFN-γ could significantly accelerate STAT1 (signal transducer and activator of transcription protein 1)-dependent degradation of the transcription factor LXRα (liver X receptor α), which can regulate cholesterol efflux by increasing the expression of ABCA1 and ABCG1 under abundance of cellular cholesterol via activation of its polyubiquitination [59], and upregulate the expression of PERK, eIIF2α, and CHOP proteins, thus contributing to ER stress and enhancing apoptotic processes [60]. TNFα might also promote ER stress-depended apoptosis in macrophages via activation of UPR pathways [61].

Autophagy is an important process in terms of cellular energy homeostasis through lysosomal degradation of cellular components, such as aggregated proteins and damaged organelles, as well as lipoproteins taken up by macrophages [62]. Autophagy can be an alternative to apoptosis under ER stress and can be beneficial to inhibiting apoptosis and inflammation, and to promoting efferocytosis and cholesterol efflux [63]. ER stress can induce autophagy by regulating PERK and IRE1-dependent pathways. It was found that knockdown of the transcription factor CCAAT/enhancer-binding protein beta (C/EBPβ) may induce the expression of autophagy-related proteins LC3A/B-II and ATG5, and at the same time, reduce phosphorylation of mammalian target of rapamycin protein (mTOR) and its gene expression in macrophages exposed to oxLDL [64]. Being a key cellular regulator of metabolism and autophagy, mTOR is tightly involved in cholesterol handling by cells. One of the pathways by which mTOR can regulate cholesterol efflux is through transcription factor EB (TFEB), which is a well-known regulator of biogenesis of lysosomes and autophagosomes. The activity and subcellular localization of this factor is regulated by mTOR. Inhibition of mTOR can increase the expression of proteins involved in cholesterol efflux from macrophages by TFEB translocation into the nucleus [64]. Known triggers of apoptosis and their action are presented in Table 2.

## 4. ER Stress and Inflammation

ER stress can participate in cellular inflammatory response through NF-κB, activator protein-1, and JNK signaling pathways, and through ROS generation. Under ER stress, IRE1 and PERK-pathways can trigger pro-inflammatory response by activating NF-κB and JNK, that are involved in regulation of pro-inflammatory genes. IRE1 binds with the scaffold protein TRAF2, which is a main component of TNF-receptor signaling. TRAF2 promotes the NF-κB release from its inhibitor IκB. TRAF2 is also responsible for ASK1-dependent activation of JNK. PERK can induce NF-κB through EIf2α. PERK/EIf2α inhibits the IKβ synthesis, which lead to nuclear translocation of NF-κB [65,66].

Altered Ca^2+^ influx in macrophages can induce production of pro-inflammatory cytokines, such as TNF-α, IL-6, and IL-1β. Furthermore, it was shown that CD36 participates in the inflammatory response by regulating membrane Ca^2+^ influx via activation of membrane Ca^2+^ channels in response to ER stress. Expression of CD36 is enhanced by a range of different cytokines. Among them are M-CSF (macrophage colony-stimulating factor) and GM-CSF (granulocyte-macrophage colony-stimulating factor) that promote growth and differentiation of macrophages, IL-1 and IL-18 that induce pro-inflammatory activation, and IL-4, which is known as anti-inflammatory cytokine inducing alternative activation of macrophages [33].

The intracellular calcium concentration varies depending on its location in the cell compartments. The cytoplasmic, nuclear, and mitochondrial Ca^2+^ concentration are equal and can reach concentrations of about 1*10^-7^ M, while the ER calcium can reach 1-8*10^-4^ M [67]. Maintenance of intracellular Ca^2+^ level is an active process performed by plasma membrane Ca^2+^ transport ATPase (PMCA), the Na^+^/Ca^2+^ exchanger (NCX), sarcoendoplasmic reticulum Ca^2+^-ATPase (SERCA) located in the ER and mitochondrial Ca^2+^ uniporter (mtCU). Intracellular Ca^2+^ oscillations play an important signaling function. Significant threshold increases in cellular Ca^2+^ concentration trigger the activation of transporters and the removal of excess intracellular Ca^2+^ in the ER and mitochondria [68].

Induction of ER stress blocks Ca^2+^ entry, leading to a reduced level of ER Ca^2+^, which in turn triggers the UPR. Reduction of ER and cytoplasmic Ca^2+^ levels is mainly determined by a decreased expression of the plasma membrane proteins Orai1, STIM1, and TRPC1, which are involved in the activation of store-operated Ca^2+^ entry [53]. Orai1 provides the main influx of Ca^2+^ in macrophages, while TRPC1, in addition to mediating of the Ca^2+^ influx, promotes their differentiation into the pro-inflammatory (M1) phenotype under inflammatory activation conditions. TRPC1 deficiency demonstrates reduced expression of inflammatory molecules in M1 macrophages, as well as the inflammatory cytokines TNF-α and IL-6 [69]. Furthermore, intracellular Ca^2+^ increase, depending on the production of nitric oxide (NO) and proinflammatory molecules, affects the ROS production [70]. Loss of TRPC1-Orai1 function induces UPR, subsequent ER stress, and subsequent cell death through apoptosis. In addition to this, Ca^2+^ deficiency enhances macrophage differentiation to the M1 phenotype and induces cytokine production [53]. Ca^2+^ release from the ER storage into the cytoplasm is triggered by activation of the phosphoinositide signaling pathway and the interaction of inositol 1,4,5-triphosphate (InsP3) with InsP3 receptors (InsP3R) on the ER membrane. The main regulator of Ca^2+^ homeostasis during ER stress is IRE1α. Inactivation of IRE1α increases the concentration of cytosolic Ca^2+^ through its release from ER storages via InsP3R, that leads to activation of the pro-apoptotic kinase ASK1, mitochondrial ROS production, mitochondrial dysfunction, and further cell death [71]. Mitochondria absorb Ca^2+^ in the ER–mitochondria membrane contact sites, which are referred to as mitochondria-associated membranes. These sites contain microdomains with voltage-dependent anion channels enriched with Ca^2+^. Then, Ca^2+^ is transferred through the outer mitochondrial membrane and mtCU to the matrix. In addition, mtCU are involved in the opening of the mitochondrial permeability transition pore (PTP), which is a key event triggering apoptotic cell death [72].

As a result of ER stress, released Ca^2+^ can induce NADPH oxidase activation in the plasma membrane, causing the transmission of ROS signals, including H_2_O_2_. In turn, H_2_O_2_ permeability of the ER membrane can further increase ER stress [73]. Not only mitochondrial OXPHOS disorders, but also oxidoreductase Ero1 and NADPH oxidase 4 in ER can serve as sources of ROS. ROS production in the ER can lead to PERK-mediated pathway UPR activation through inhibition of protein tyrosine phosphatase 1B, as well as activation of Nrf2 via IRE1 [74]. The mitochondrial ROS accumulation can further enhance the release of Ca^2+^ from the ER and increase UPR. Thus, Ca^2+^ release from the intracellular storage and uptake by mitochondria, the production of ROS and UPR together cause the activation of Ca^2+^-dependent protein kinases, as well as JNK and NF-κB, which leads to apoptosis and inflammatory response [75].

The relationship between ER stress activation and mitochondrial dysfunction in macrophages was established under inflammatory conditions [76]. Activation of the PERK/CHOP pathway by LPS promotes IL-23 expression, which is enhanced by mitochondrial dysfunction. Thus, mitochondrial dysfunction in combination with ER stress can enhance IL-23 production in macrophages.

Angiotensin II (Ang II) is a pro-inflammatory cytokine that contributes to atherogenesis [77]. By interacting with the angiotensin II receptor type 1, Ang II activates the AMPK/p38MAPK/monocyte chemotactic protein-induced protein 1 (MCPIP1)/ER stress signaling pathway, which leads to ER stress-mediated macrophage apoptosis.

The new Akt/XBP1 signaling pathway induced by IL15 has recently been identified, which enhances effector functions and immune cell survival by activating the anti-apoptotic protein Bcl-2 [78].

A special contribution to the development of pro-inflammatory response in macrophages is made by NOD-like receptor protein 3 (NLRP3) inflammasome, the induction of which occurs because of the activation of TLRs on the cell surface. This leads to the expression of the transcription factor NF-κB, which promotes the formation of NLRP3, and precursors of IL-1β and IL-18 [79,80]. ER stress induces the NLRP3 formation in different cells types, including macrophages, through the activation of the IRE1α and PERK pathways [7]. Mitochondria-associated membranes, which serve as a scaffold for crosstalk between the ER and mitochondria, can also serve as a platform for NLRP3 inflammasome assembly [81]. It was shown that one of the key regulators of inflammasome activation in macrophages is serine-threonine kinase receptor-interacting protein 1 (RIP1), a protein belonging to the RIP family, which serves as a regulator of cell survival and death in response to various stress stimuli [82]. Activation of RIP1 mediates the NLRP3 assembly, which promotes maturation of pro-IL-1β and pro-IL-18 via caspase-1, and secretion of IL-1β and IL-18 by cells. In addition, induction of NLRP3 formation requires apoptosis signal-regulating kinase 1 (ASK1), a member of the mitogen-activated protein kinase (MAPK) family, which can be activated due to TLR activation and ER stress [83,84]. It was shown that exposure to oxLDL can reduce the expression of ABCA1 and ABCG1 and induce NLRP3 activation. This can result in subsequent cell death by apoptosis due to ASK1 overexpression and ER stress in endothelial cells [84]. Furthermore, protease cathepsin B, which is a member of the lysosomal cysteine cathepsin family, can be involved in the regulation of NLRP3 inflammasome activation, and was shown to be active during monocyte differentiation into macrophages [85,86]. In case of cathepsin B deficiency, the IL-1β production does not occur in macrophages. It was suggested that cathepsin B can translocate from the lysosomes to the ER to interact with inactivated NLRP3 [86]. Thioredoxin-interacting protein (TXNIP), including macrophages, was also identified as a new regulator of NLRP3 induction. Activation of ER stress by endogenous lipids in cells leads to increased expression of the markers UPR, TXNIP, NLRP3, and IL-1β, which indicates the development of pro-inflammatory response in macrophages via the ER/TXNIP/NLRP3 stress pathway [87]. All these processes lead to increased cytokine production by macrophages upon ER stress (Table 3).

## 5. Relationship between Cholesterol Accumulation, ER Stress, and Pro-Inflammatory Response

**Figure 1:**
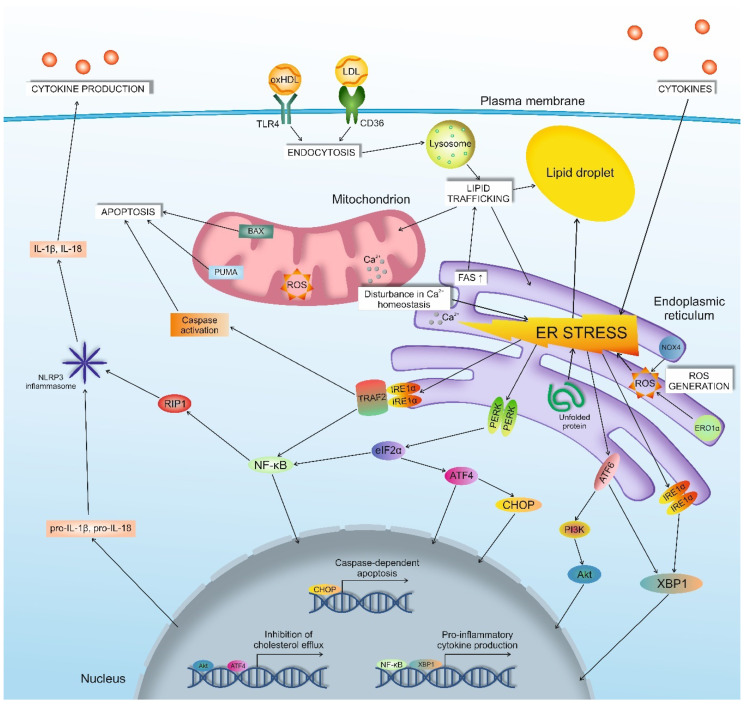
Relationship between cholesterol accumulation, ER stress, and pro-inflammatory response. Pro-inflammatory cytokines, atherogenic LDL, oxHDL may cause accumulation of unfolded protein, disturbances in calcium homeostasis, ROS generation, and/or exceeding lipid accumulation in ER that can trigger the unfolded protein response, where the key players are PERK, IRE1α, and ATF6. PERK, IRE1α, and ATF6 mediated downstream pathways, which can launch pro-inflammatory response, apoptosis, reduction of cholesterol efflux, or/and increase of cholesterol uptake, which may lead to foam cell formation via lipid droplet accumulation. ER—endoplasmic reticulum, FAO—fatty acid oxidation, FAS—fatty acid synthesis, ROS—reactive oxygen species.

The observations listed above allow suggesting the existence of a relationship between cholesterol accumulation, ER stress, and pro-inflammatory response (Figure 1). This relationship is possible, because ER apparently is the central organelle, through which lipid homeostasis is maintained (Table 4).

One of the causes of the ER stress development in macrophages is the excessive free cholesterol accumulation in the ER membrane. In atherosclerosis, macrophages actively uptake oxidized and other modified LDL penetrating into the intima of blood vessels [88]. Activated macrophages internalize the extracellular lipids, which leads to their excessive intracellular accumulation and triggering ER stress-mediated IRE1α and PERK signaling pathways. Moreover, lipid-loaded macrophages induce both apoptosis and secretion of TNF-α and IL-6 as a result of exceeded amount of free cholesterol in ER [89]. Specifically, IKK/NF-κB and JNK1/2 pathways are involved in production of both cytokines, whereas MKK3/p38 pathway mediates TNF-α production, and the branch of UPR pathway, CHOP/Erk1/2, is required for IL-6. IRE1α/TRAF2 and PERK/STAT3 pathways may provide a link between ER stress/UPR and inflammation [89,90]. IRE1α signaling is active in both splicing of XBP1 mRNA and an alternative IRE1α/TRAF2 pathway [91]. The IRE1α/TRAF2 complex can translocate NF-κB and recruit the expression of NF-κB-dependent cytokines [92]. PERK/elF2α pathway induces the CHOP, which can lead to IL-23 expression. Under ER stress, PERK/STAT3 signaling induces inflammatory response by releasing IL-6 and IL-8 [93,94].

Macrophage activation occurs through the DAMP (damage-associated molecular patterns) and PAMP (pathogen-associated molecular patterns) interaction with TLR [95]. TLR activation leads to the IRE1α/XBP1 signaling pathway (UPR activation) induction and subsequent production of the pro-inflammatory cytokines IL6, CCL2, and TNF-α. TLR activation can not only cause the IRE1α and PERK signaling pathways induction, but also regulate their suppression via PP2A, which mediates dephosphorylation of IRE1α and eIF2 [96]. IRE1α, as well as PERK, regulates the activation of GSK3 kinases. GSK3 can suppress activation of XBP1 and regulate the expression of several pro-inflammatory cytokines, such as IL-6, IL-1β, TNF-α, IL-10 [22]. Since the IRE1α/XBP1 pathway regulates *TNF* gene transcription, activation of IRE1α/GSK3β can reduce its expression [96]. Inhibition of the PERK/eIF2/ATF4 pathway in macrophages did not cause a reduction in IL6 expression in macrophages under ER stress, which may indicate its insignificant role in the pro-inflammatory cytokine production process, and their possible contribution to the IRE1α/XBP1 pathway [97]. Thus, ER stress can directly induce the inflammatory response.

However, inflammatory mediators, such as IL-1β, IL-6, and TNF-α, in turn, can have direct or indirect impact on the ER, provoking ER stress via ROS generation and Ca^2+^ perturbation [94]. ROS production is a known ER stress trigger, which activates PERK and further anti-oxidative stress response [98]. Elevated ROS levels and Ca^2+^ disruption can induce ER stress resulting in activation of all three UPR branches, as well as STAT3 and TRAF2-mediated signaling pathways that further aggravate the inflammatory response and ER stress [99].

Moreover, interleukins may modulate the expression of scavenger receptors and ABCA1. Thus, IL-6 can increase expression of SR-AI and ABCA1, that may lead to foam cell formation or may attenuate lipid accumulation, depending on which receptor is more activated [100,101]. IL-1β can increase the expression of LOX-1 and decrease the expression of ABCA1, which promotes foam cell formation [102]. IL-17A can also up-regulate LOX-1 expression and enhance foam cell formation [103]. IL-8 suppresses ABCA1 expression and cholesterol efflux via activation of miR183 [104]. IL-32 may attenuate ABCA1 expression through PPARγ/LXRα/ABCA1 pathway, which abolishes cholesterol efflux and provokes lipid accumulation [105]. IL-18 might activate CD36 expression in macrophages treated with oxLDL [106]. IL-34 can mediate foam cell formation by increasing CD36 expression through p38 MAPK pathway [107]. Interferon-γ, in turn, may up-regulate CD36 expression through activation of JAK-STAT pathway [108,109]. Furthermore, it was shown that TNF-α can enhance lipid accumulation by up-regulation of both SR-AI and LOX-1 [110].

The crosstalk between ER stress and inflammation has been revealed through several observations. Activated by TLRs, IRE1α/TRAF2 pathway promotes NF-κB mediated production and secretion of pro-inflammatory cytokines, such as TNF-α, IL-1β. Intercellular signaling of TNF-α and IL-1β induces elevated intracellular ROS levels and disturbance in Ca^2+^ homeostasis. Furthermore, CD36 is one of the possible components that can explain interaction between lipid accumulation, inflammation, and ER stress. CD36 and TLR4 activate NF-κB pathway, which leads to inflammation in macrophages [111]. Atherogenic stress factors such as oxLDL, oxidized phospholipids, lipoproteins, and fatty acids, can trigger oxidative burst through the CD36/TLR2/TLR6 pathway with subsequent apoptosis of cholesterol-overloaded foam cells, which promotes a pro-inflammatory response from macrophages [112]. Furthermore, CD36 expression is up-regulated by pro-inflammatory cytokines. The way by which CD36 promotes TLRs activation is not clearly understood. It was suggested that such activation may be mediated by the Src-family pathway [112]. It can be concluded, however, that CD36 acts as a central regulator of NLRP3 inflammasome activation. This relationship is responsible for cleavage of pro-IL-1β and pro-IL-18 [113].

In macrophages, excessive TNF-α signaling can promote the expression of miR-21, which acts according to the feedback principle and alleviates inflammation [114]. It was shown that reduced expression of miR-21 in macrophages with impaired cholesterol efflux initiate increased expression of mitogen-activated protein kinase kinase 3 (MKK3) [115]. This, in turn, leads to activation of the p38MAPK/CHOP and JNK signaling pathways, causing ER stress-induced apoptosis in macrophages. These results emphasize the importance of miR-21 in the regulation of macrophage apoptosis and lipid metabolism [115].

## 6. Conclusions

Macrophages play an important role in atherosclerosis because of the conversion to foam cells and the production of pro-inflammatory cytokines. An important role in these processes is played by the ER, which normally maintains lipid, protein, and calcium homeostasis within the cell. Pathological conditions can induce ER stress, which is both a consequence and a cause of disturbance in cellular homeostasis. Thus, disturbance of lipid homeostasis impacts other cell functions, as well as secretion of pro-inflammatory cytokines, that promote stress in other cells, propagating the pathological process. That leads to a vicious circle formation. It is possible that the nature of the initial trigger of the ER stress, be it cytokine signaling, presence of modified LDL, or release of PAMPs, is less relevant for the disease development than the pathogenic cascade that follows, since this cascade can transform to a self-sustaining chronic condition. Despite all the advances in the study of ER stress, the complex relationships of the processes involved still need to be described in detail. Moreover, the exact causes the foam cell formation are not fully understood. They can include either modified LDL or cytokines that provoke ER stress, which mediates up-regulation of CD36 and subsequently increases the uptake of modified LDL. Whether the accumulation of lipids or the pro-inflammatory stimulation of macrophages is initial is to be clarified by the future studies.

## Figures and Tables

**Table 1:** Macrophage lipid accumulation under endoplasmic reticulum stress.

Triggers of Foam Cells Formation	Effects
Modified Atherogenic LDL Particles	• oxLDL is bound and internalized by CD36 and TLR4; • oxLDL activates the ER stress through PERK, ATF6, and IRE1-mediated UPR pathways.
Reduced Cholesterol Efflux	• downregulation of miR-21 expression and subsequent activation of p38MAPK/CHOP and JNK signaling pathways inhibits expression of ABCG1 and SR-BI; • overexpression of miR-33, leading to activation of PI3K/Akt signaling pathway by ATF6, inhibits the ABCA1 and ABCG1 expression. • Activated PERK/eIF2α/ATF4 and IRE1α/XBP1 signaling pathways can suppress the ABCA1 expression.
Alterations in Lipid Biosynthesis	• ATF6 regulates SREBP2, that can lead to an increased expression of HMG-CoA reductase and HMG-CoA synthase; • PERK regulates the expression of SREBP1 and FAS through the eIF2-dependent manner.

**Table 2:** Role of the endoplasmic reticulum stress in activation of apoptotic cell death pathways.

Apoptosis Triggers	Effects
Excessive Lipid Accumulation	High concentrations of free cholesterol, oxysterols, and oxLDL cause prolonged ER-stress, which induces the macrophage apoptosis via activation of CHOP through IRE1α/JNK/MAPK, IRE1α/XBP1, ATF6/XBP1, and PERK/eIF2α/ATF4 pathways
Inflammatory Processes	IFN-γ accelerates STAT1-dependent degradation of the transcription factor LXRα and upregulate the PERK/eIIF2α/CHOP pathway, thus contributing to ER stress and enhancing apoptotic processes.
Oxidized HDL	Binding of oxHDL to TLR4 can induce macrophage apoptosis by activation an ER stress/CHOP pathway through enhanced oxidative stress.
Ca^2+^	Increased calcium concentration in ER may lead to absorption of Ca^2+^ by mitochondria, which can lead to PTP opening, releasing of apoptotic factors and subsequent inducing of apoptosis.

**Table 3:** Macrophage pro-inflammatory response during endoplasmic reticulum stress.

The Cause of the Pro-Inflammatory Cell Response	Cytokines Involved
Change in the calcium influx	TNF-α, IL-6, and IL-1β
Excessive lipid accumulation	TNF-α and IL-6
Activation of PERK/CHOP pathway, which is enhanced by mitochondrial dysfunction	IL-23
Activation of PERK/STAT3 pathway	IL-6 and IL-8
Activation of IRE1α/XBP1 pathway	IL-6, CCL2, and TNF-α
Activation of TLRs on the cell surface, leading to the formation of NLRP3 inflammasome	IL-1β and IL-18

**Table 4:** Common stress factors for ER stress, lipid accumulation and inflammation and complex relationship between these processes.

Stress Factors	Relationship
TLR activation	TLR activation leads to the IRE1α/XBP1 signaling pathway induction and subsequent production of the pro-inflammatory cytokines IL6, CCL2, TNF-α. Also, TLR activates the NF-κB that leads to NLRP3 inflammasome activation and production of IL-1β and IL-18.
CD36	CD36/TLR2/TLR6 pathway activate NF-κB, resulting in apoptosis of endoplasmic reticulum-stressed cholesterol-overloaded foam cells, which promotes pro-inflammatory response. CD36 act as another regulator of NLRP3 inflammasome activation.
Lipid accumulation	Cholesterol accumulation triggers IRE1α and PERK signaling pathways, that can induce both apoptosis and secretion of TNF-α and IL-6.
Ca^2+^ and ROS	Ca^2+^ deficiency activates UPR, enhances macrophage differentiation by the pro-inflammatory M1 phenotype, induces cytokine production, and can lead to apoptosis. Elevated ROS levels and Ca^2+^ disruption can induce activation all UPR signaling pathways, as well as STAT3 and TRAF2-mediated pathways that aggravate the inflammatory response and ER stress.
Mitochondrial dysfunction during inflammation	Activation of the PERK/CHOP pathway promotes IL-23 expression, which is enhanced by mitochondrial dysfunction.
Proinflammatory agents	Cytokines can activate ER stress through perturbation in ROS and Ca^2+^ homeostasis; can promote lipid accumulation by upregulating expression of scavenger receptors (CD36, LOX-1, SR-AI) or by diminishing the ABCA1-depending cholesterol efflux.
